# Supplementary material for: RNA N6-methyladenosine modification-based biomarkers for absorbed ionizing radiation dose estimation
Source: Nat Commun. 2023 Oct 30;14:6912. doi: 10.1038/s41467-023-42665-w (PMC10616291; doi:10.1038/s41467-023-42665-w)
Supplement: Supplementary file 1 — Supplementary Information [file 41467_2023_42665_MOESM1_ESM.pdf]

Supplementary Figures

Supplementary Fig. 1

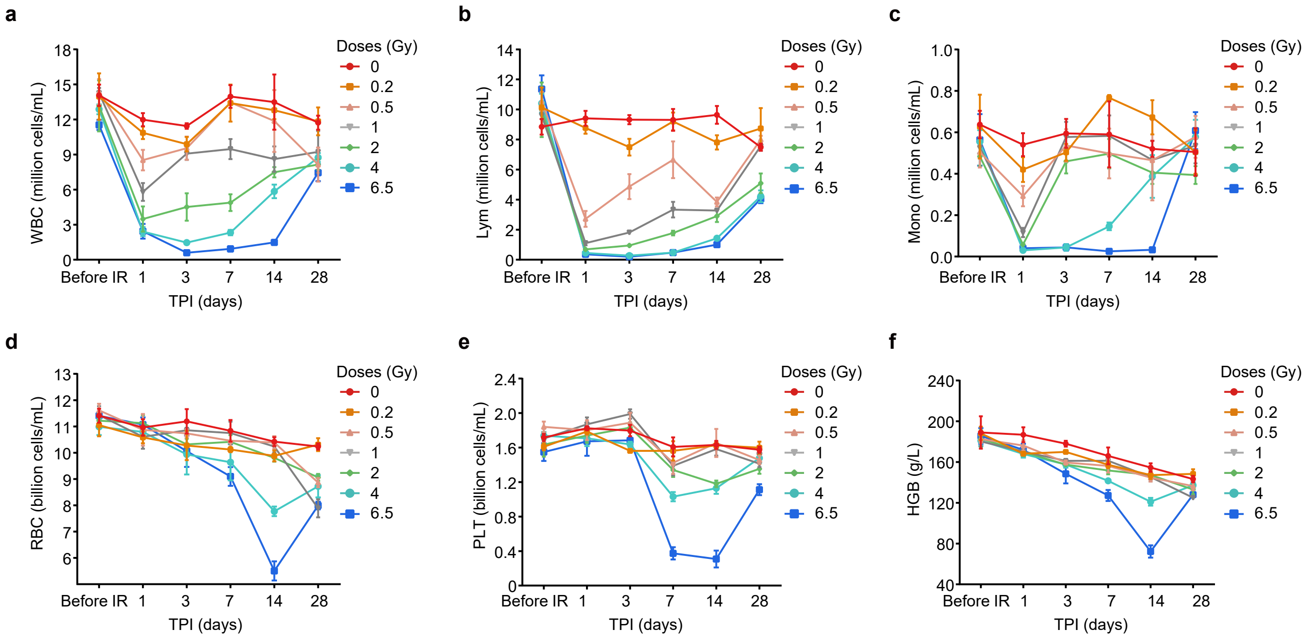

Supplementary Fig. 2

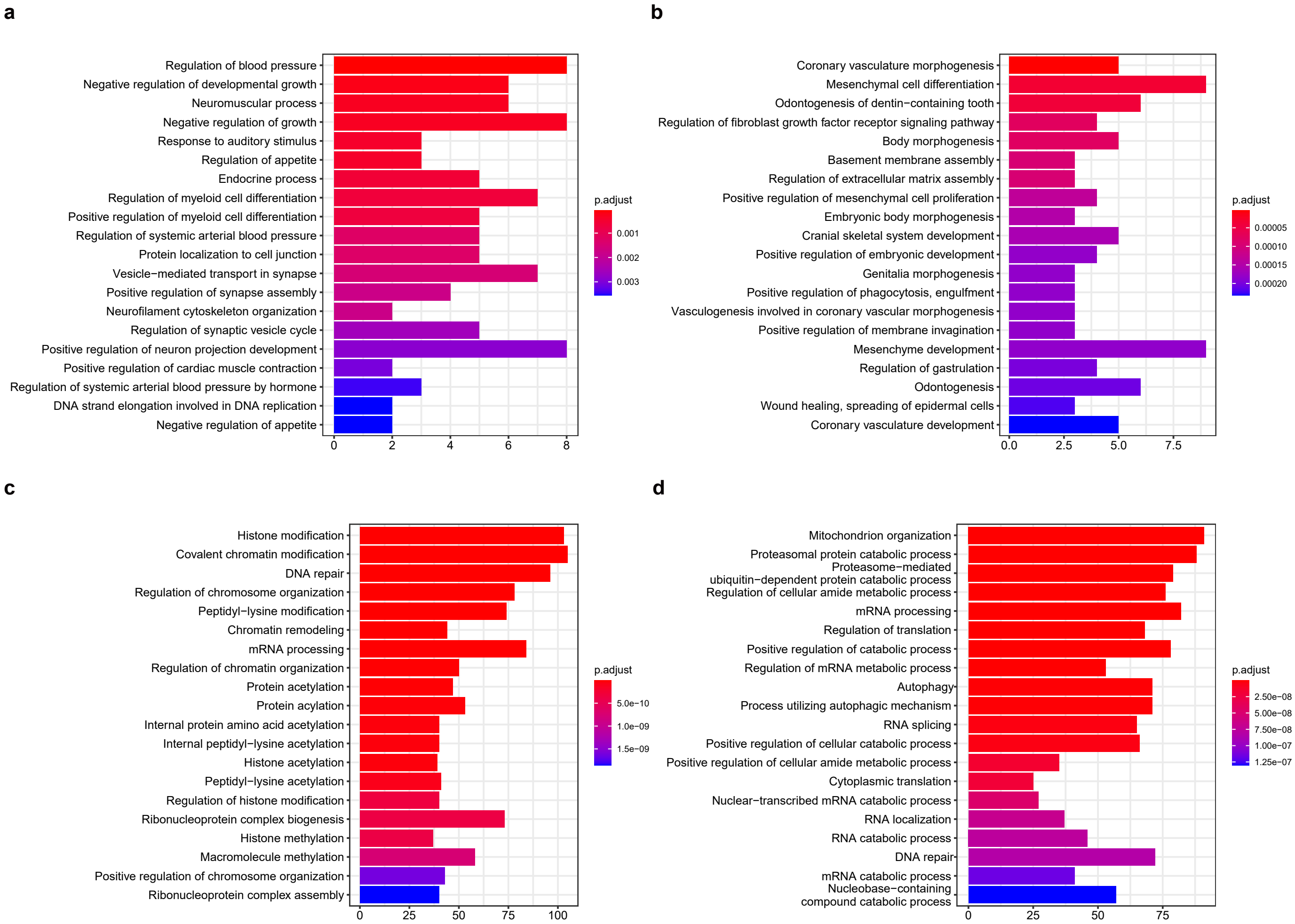

Supplementary Fig. 3

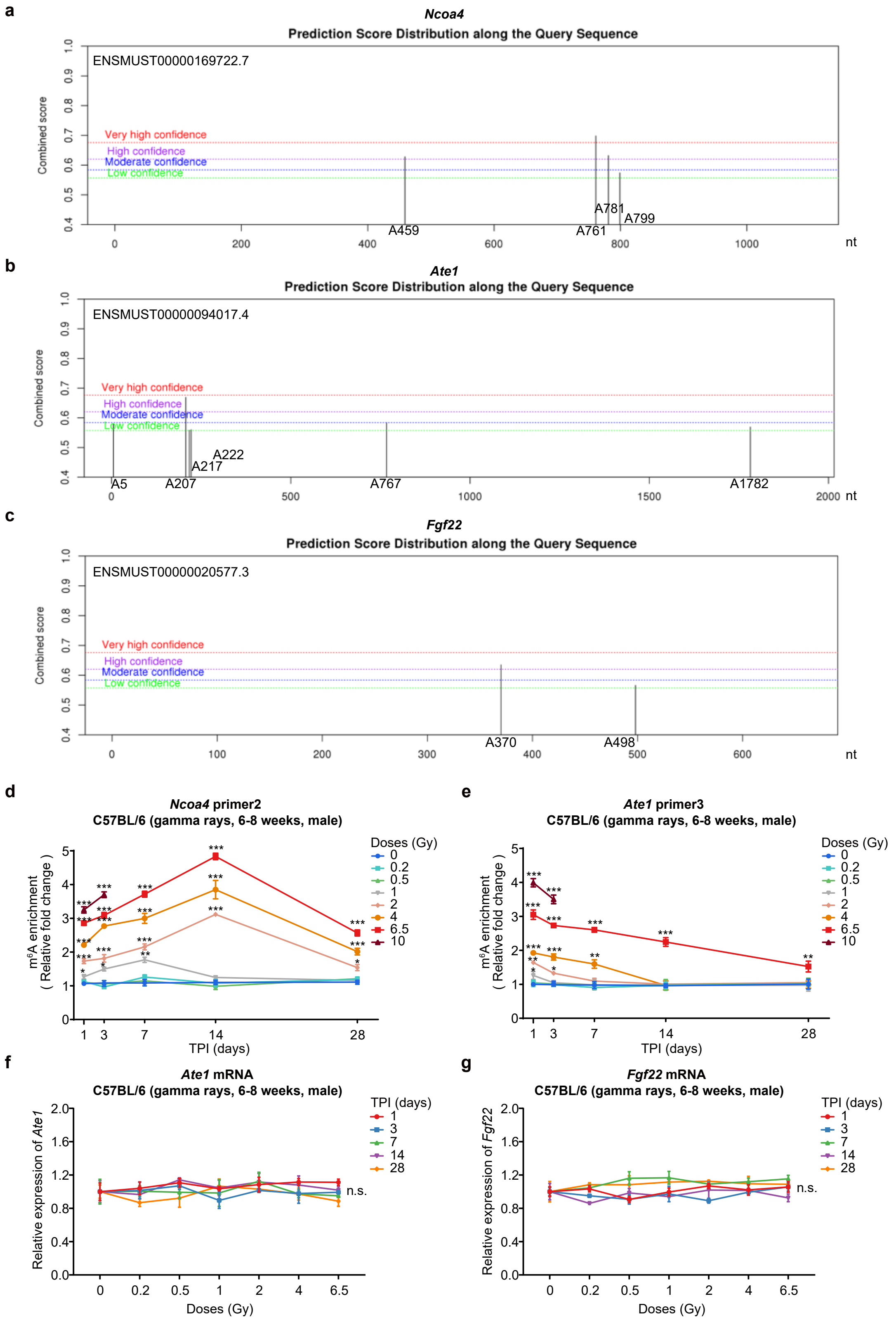

Supplementary Fig. 4

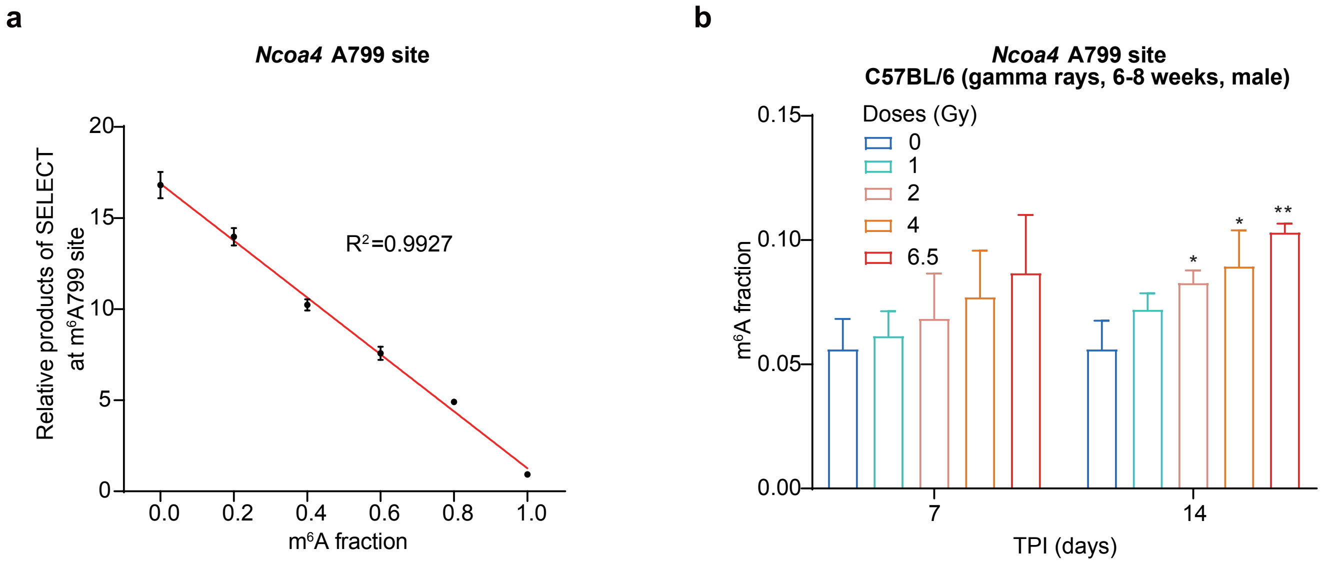

Supplementary Fig. 5

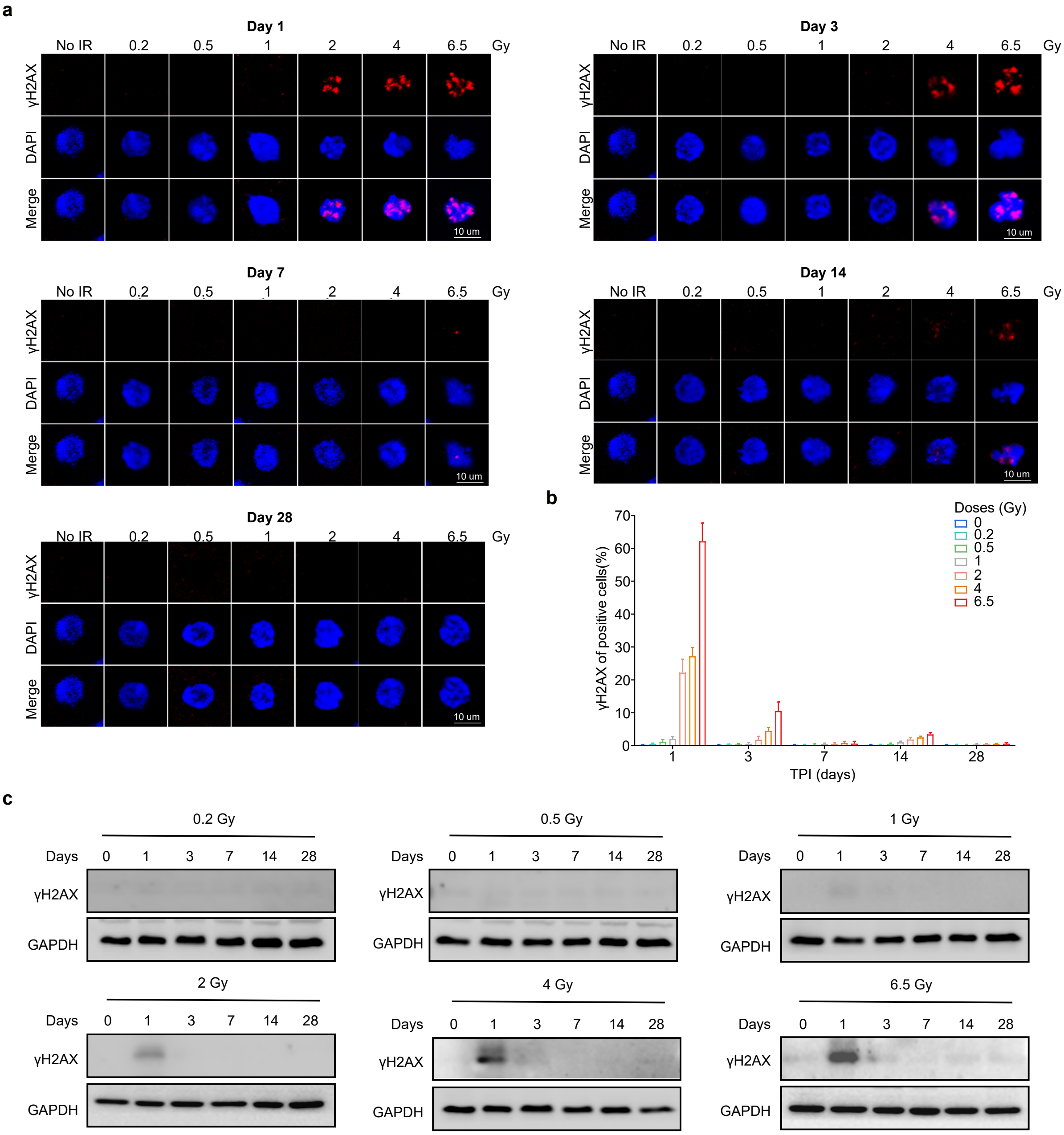

Supplementary Fig. 6

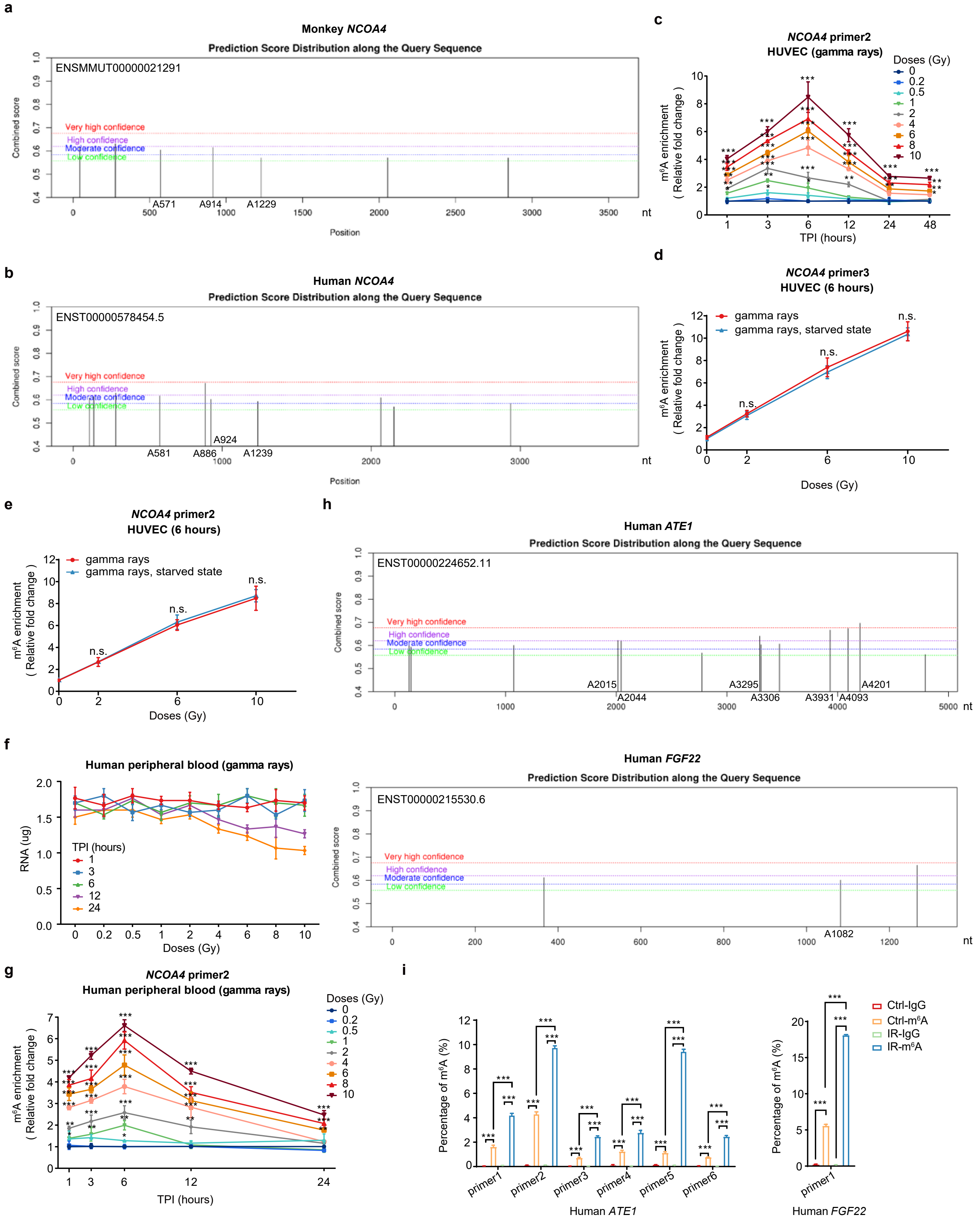

Supplementary Fig. 7

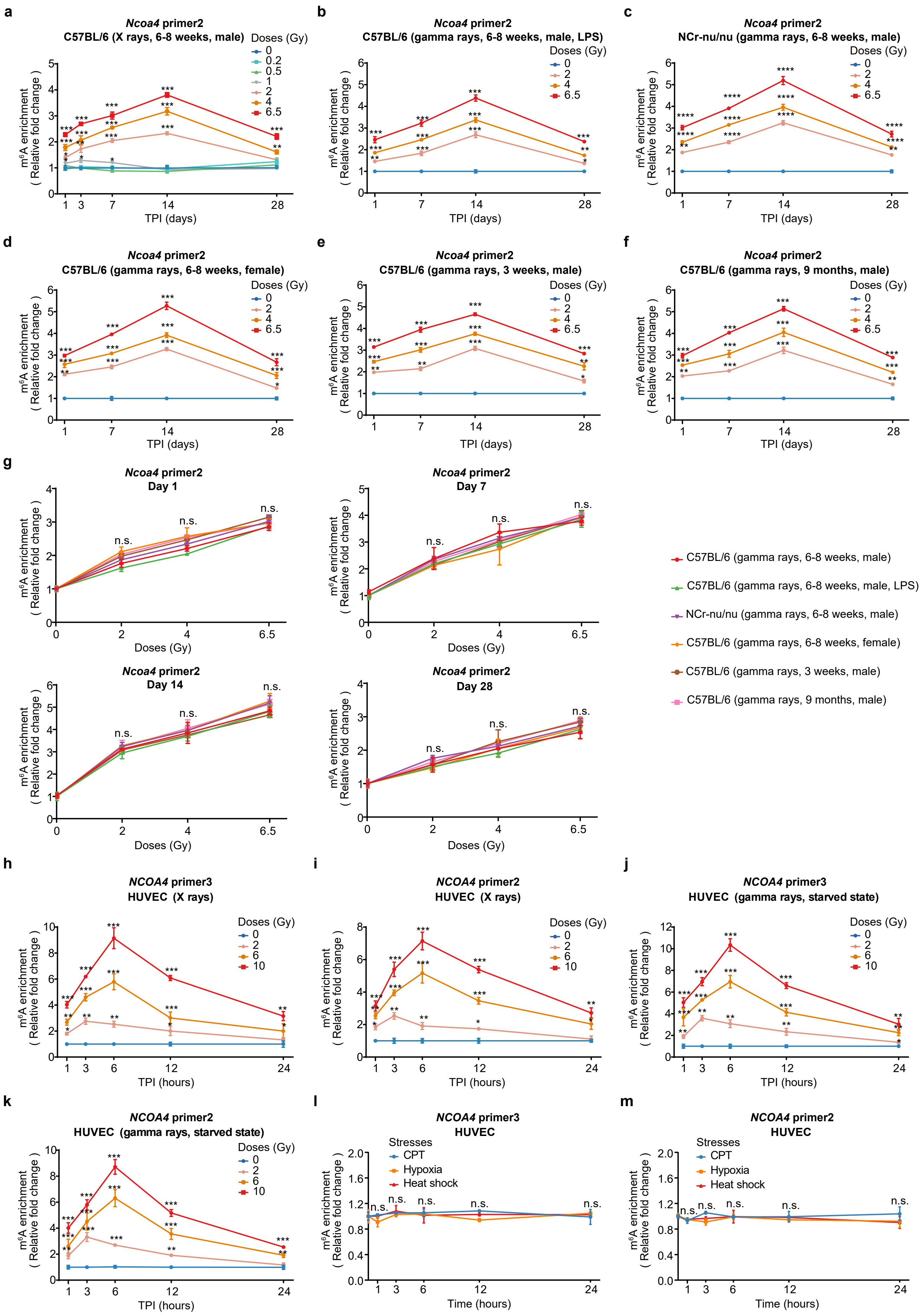

Supplementary Fig. 8

a

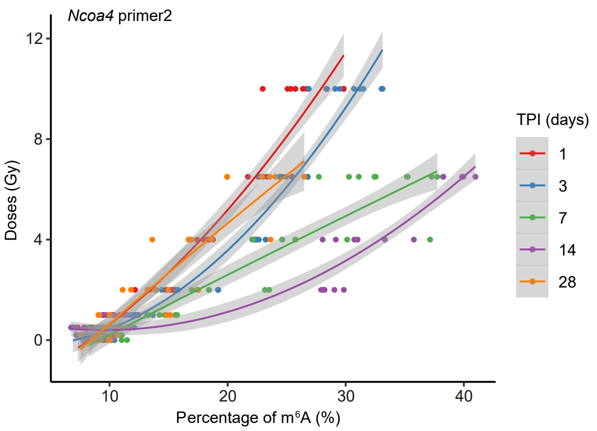

TPI (days)	Intercept	*b* (*x*)	*b* (x^2)	R^2	*p*-value
1	-2.222	0.198	8.58E-3	0.945	1.84E-28
3	-0.134	-0.071	0.013	0.952	2.17E-32
7	-2.220	0.245	-2.27E-4	0.897	2.12E-22
14	0.993	-0.125	6.58E-3	0.941	1.07E-24
28	-3.589	0.435	-1.12E-3	0.847	1.33E-16

b

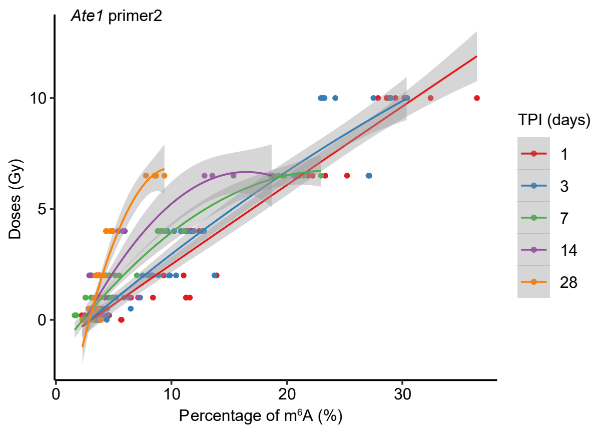

TPI (days)	Intercept	*b* (*x*)	*b* (x^2)	R^2	*p*-value
1	-1.117	0.363	-1.77E-4	0.937	1.08E-30
3	-1.320	0.455	-2.75E-3	0.915	4.31E-29
7	-1.543	0.693	-0.015	0.939	7.50E-24
14	-2.612	1.126	-0.034	0.768	8.92E-13
28	-6.790	2.744	-0.138	0.852	4.20E-16

c

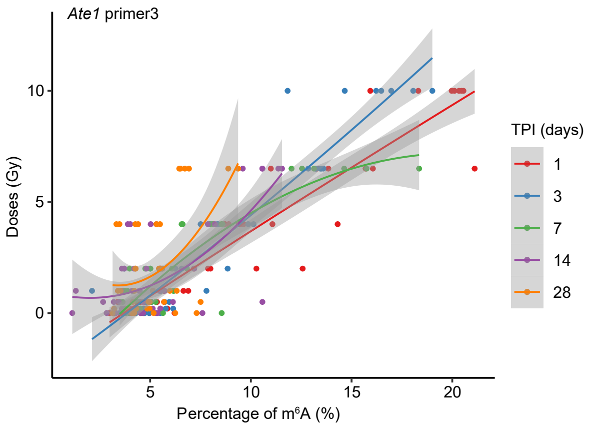

TPI (days)	Intercept	*b* (*x*)	*b* (x^2)	R^2	*p*-value
1	-2.193	0.598	1.01E-3	0.888	5.66E-24
3	-2.574	0.652	4.58E-3	0.903	1.65E-23
7	-3.379	1.046	-0.026	0.818	3.60E-15
14	0.933	-0.248	0.062	0.523	2.50E-07
28	3.028	-1.050	0.154	0.27	2.48E-3

d

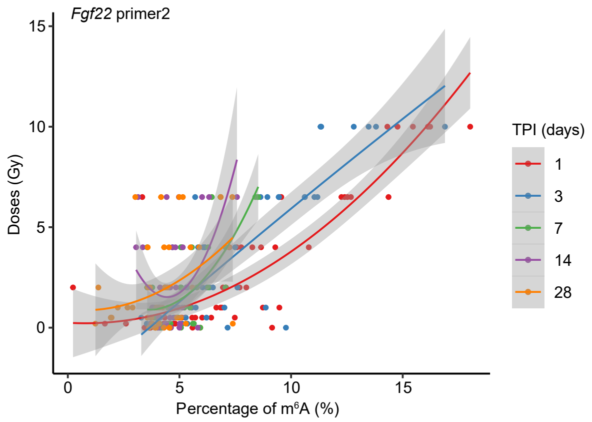

TPI (days)	Intercept	*b* (*x*)	*b* (x^2)	R^2	*p*-value
1	0.245	-0.065	0.042	0.791	4.49E-18
3	-3.538	0.981	-3.53E-3	0.735	3.71E-15
7	4.353	-1.885	0.258	0.561	6.97E-8
14	15.384	-6.229	0.700	0.261	3.70E-3
28	0.912	-0.119	0.082	0.159	0.034

e

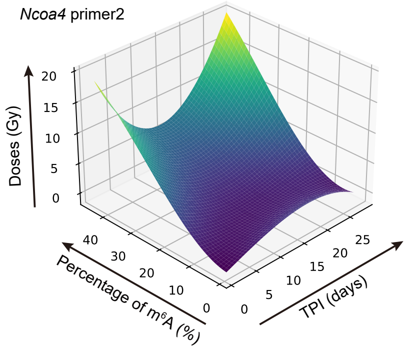

Intercept	*b* (*x*)	*b* (x^2)	*b* (x^3)
-1.640	0.410	-6.60E-4	-4.11E-4
***b* (*y*)**	***b* (*xy*)**	***b* (x^2y)**	***b* (y^2)**
0.070	-0.065	1.98E-3	0.021
***b* (xy^2)**	***b* (y^3)**	**R^2**	***p*-value**
1.70E-4	-2.54E-4	0.924	7.09E-129
