# Supplementary material for: RNA N6-methyladenosine modification-based biomarkers for absorbed ionizing radiation dose estimation
Source: Nat Commun. 2023 Oct 30;14:6912. doi: 10.1038/s41467-023-42665-w (PMC10616291; doi:10.1038/s41467-023-42665-w)
Supplement: Supplementary file 2 — Reporting Summary [file 41467_2023_42665_MOESM2_ESM.pdf]

nature portfolio

Corresponding author(s): Gangqiao Zhou

Last updated by author(s): Aug 30, 2023

Reporting Summary

Nature Portfolio wishes to improve the reproducibility of the work that we publish. This form provides structure for consistency and transparency in reporting. For further information on Nature Portfolio policies, see our Editorial Policies and the Editorial Policy Checklist.

Statistics

For all statistical analyses, confirm that the following items are present in the figure legend, table legend, main text, or Methods section.

n/a	Confirmed	
☐	☒	The exact sample size (*n*) for each experimental group/condition, given as a discrete number and unit of measurement
☐	☒	A statement on whether measurements were taken from distinct samples or whether the same sample was measured repeatedly
☐	☒	The statistical test(s) used AND whether they are one- or two-sided<br>*Only common tests should be described solely by name; describe more complex techniques in the Methods section.*
☐	☒	A description of all covariates tested
☐	☒	A description of any assumptions or corrections, such as tests of normality and adjustment for multiple comparisons
☐	☒	A full description of the statistical parameters including central tendency (e.g. means) or other basic estimates (e.g. regression coefficient) AND variation (e.g. standard deviation) or associated estimates of uncertainty (e.g. confidence intervals)
☐	☒	For null hypothesis testing, the test statistic (e.g. F, t, r) with confidence intervals, effect sizes, degrees of freedom and P value noted<br>*Give P values as exact values whenever suitable.*
☒	☐	For Bayesian analysis, information on the choice of priors and Markov chain Monte Carlo settings
☒	☐	For hierarchical and complex designs, identification of the appropriate level for tests and full reporting of outcomes
☒	☐	Estimates of effect sizes (e.g. Cohen's *d*, Pearson's *r*), indicating how they were calculated

Our web collection on statistics for biologists contains articles on many of the points above.

Software and code

Policy information about availability of computer code

Data collection	The white blood cell (WBC), lymphocyte (Lym), monocyte (Mono), red blood cell (RBC), and platelet (PLT) counts, as well as the hemoglobin (HGB) concentration were obtained using the automated URIT-5160Vet Hematology Analyzer (URIT Medical Electronic, China). Digital images were obtained using a fluorescence microscope (Nikon, Japanese) and fluorescence intensity was analyzed by imageJ. Transcriptome-wide mRNAs expressions and m6A modification levels were quantified using the Arraystar Mouse RNA Epi-transcriptomic Microarray (8×60K, Arraystar, Rockville, MD, USA) based on the Arraystar's standard protocols.
Data analysis	Agilent Feature Extraction software (version 11.0.1.1) was used to analyze the acquired array images. An unsupervised clustering analysis was performed by using timeclust function in the TCseq R package (v.1.14.0). Statistical analyses were performed by Prism 8.0 software (GraphPad, USA). Polynomial regression models were used for the estimation of radiation doses. To construct radiation doses estimation models in a given day post irradiation both in mice and humans, we employed a two-order polynomial regression model to fit the actual dose of exposure with the observed m6A levels of NCOA4 in PBMCs. To construct an integrated model that takes both m6A levels and time post irradiation as input for the doses estimation in mice, a binary three-order polynomial regression model was introduced. The fitness between the actual and estimated doses were evaluated using the coefficient of determination (R2). Besides, 100 times repeated five-fold cross-validation procedure and receiver operating characteristic (ROC) analyses were used to assess the performance of the estimation model on the prediction of absorbed irradiation dose. The two-order polynomial regression models were implemented using R language (v4.0.3), the binary three-order polynomial regression model was implemented using the "NumPy" package in Python (v3.8), and the ROC analysis was performed using the "ROCR" R package.

For manuscripts utilizing custom algorithms or software that are central to the research but not yet described in published literature, software must be made available to editors and reviewers. We strongly encourage code deposition in a community repository (e.g. GitHub). See the Nature Portfolio guidelines for submitting code & software for further information.

Data

Policy information about availability of data

All manuscripts must include a data availability statement. This statement should provide the following information, where applicable:

- Accession codes, unique identifiers, or web links for publicly available datasets
- A description of any restrictions on data availability
- For clinical datasets or third party data, please ensure that the statement adheres to our policy

The raw and processed mRNA expression and m6A modification datasets generated by Arraystar Mouse RNA Epi-transcriptomic Microarray in stages I and II mice TBI experiments have been deposited in the GEO database under accession code GSE225404 and GSE225405. The MeRIP-PCR and SELECT data of mice, monkey and human generated in this study are provided in the Supplementary Data file.

Research involving human participants, their data, or biological material

Policy information about studies with human participants or human data. See also policy information about sex, gender (identity/presentation), and sexual orientation and race, ethnicity and racism.

Reporting on sex and gender	Sex or gender were not considered in our study involving human participants.
Reporting on race, ethnicity, or other socially relevant groupings	We did not collect any information related to race, ethnicity or other socially relevant groupings.
Population characteristics	The ages of healthy volunteers range from 23 to 37, with an average age of 28.4; The ages of cancer patients receiving radiotherapy range from 31 to 78, with an average age of 56.9; The ages of radiation workers range from 24 to 56, with an average age of 35. See Supplementary Data 4 for a detailed characteristics of the population.
Recruitment	Three human cohorts were recruited in this study (Supplementary Data 4). The first is the acute radiation-exposure cohort, which consists of 33 cancer patients (including cervical, uterine, and vaginal cancers) who were scheduled to receive local radiotherapy (TOMO Therapy Hi·Art; Accuray, USA), and were randomly recruited from the First Medical Center of Chinese PLA General Hospital (Beijing, China) between April and September in 2022. The second is the non-radiation-exposed background cohort, which consists of 16 healthy volunteers without known irradiation exposure who were randomly recruited between June and July in 2022 from the Beijing Institute of Radiation Medicine (Beijing, China). The third is the radiation-occupational-exposure cohort. A total of six operators of radioactive source, who involved in the operation of Cobalt-60 (60Co) radioactive sources or engaged in radiotherapy occupations, were recruited in July 2022 from the Beijing Institute of Radiation Medicine (Beijing, China) and the Department of Radiation Oncology in the First Medical Center of Chinese PLA General Hospital (Beijing, China), respectively.
Ethics oversight	This study was performed with the approval of the Medical Ethical Committee of Beijing Institute of Radiation Medicine (Beijing, China) and the Department of Radiation Oncology in the First Medical Center of Chinese PLA General Hospital (Beijing, China), and in accordance with the Council for International Organizations of Medical Sciences (CIOMS).

Note that full information on the approval of the study protocol must also be provided in the manuscript.

Field-specific reporting

Please select the one below that is the best fit for your research. If you are not sure, read the appropriate sections before making your selection.

☒ Life sciences ☐ Behavioural & social sciences ☐ Ecological, evolutionary & environmental sciences

For a reference copy of the document with all sections, see nature.com/documents/nr-reporting-summary-flat.pdf

Life sciences study design

All studies must disclose on these points even when the disclosure is negative.

Sample size	A minimum of five mice were used in each group in our study. The sample size was calculated based on the expected Mean and Standard Deviation (SD) values for each experiment.
Data exclusions	No data has been excluded for the analyses.
Replication	A minimum of five mice were used as biological replicates in each group in our study. We confirmed these replication were successful by measurement of body weight, hemogram, etc.
Randomization	All the mice were allocated into groups randomly.
Blinding	Investigators were blinded to group allocation during data collection and analysis.

Reporting for specific materials, systems and methods

We require information from authors about some types of materials, experimental systems and methods used in many studies. Here, indicate whether each material, system or method listed is relevant to your study. If you are not sure if a list item applies to your research, read the appropriate section before selecting a response.

Materials & experimental systems

n/a	Involved in the study	
☐	☒	Antibodies
☐	☒	Eukaryotic cell lines
☒	☐	Palaeontology and archaeology
☐	☒	Animals and other organisms
☒	☐	Clinical data
☒	☐	Dual use research of concern
☒	☐	Plants

Methods

n/a	Involved in the study	
☒	☐	ChIP-seq
☒	☐	Flow cytometry
☒	☐	MRI-based neuroimaging

Antibodies

Antibodies used	Anti-m6A antibody (56593; Cell Signaling Technology, USA) or normal IgG (2729s; Cell Signaling Technology, USA) were used for MeRIP-qPCR experiments.
Validation	Antibodys have been validated on the manufacture's websites:<br>https://www.cellsignal.com/products/primary-antibodies/n6-methyladenosine-m6a-d9d9w-rabbit-mab/56593<br>https://www.cellsignal.com/products/primary-antibodies/normal-rabbit-igg/2729

Eukaryotic cell lines

Policy information about cell lines and Sex and Gender in Research

Cell line source(s)	Human umbilical vein endothelial cells (HUVECs) were obtained from the China Center for Type Culture Collection (CCTCC; Wuhan City, China). The peripheral blood mononuclear cells (PBMCs) were isolated by density gradient centrifugation using the PBMC isolation kit (TBD sciences, China) according to the manufacturer's instructions.
Authentication	The identity of the HUVEC has been authenticated by its provider: the China Center for Type Culture Collection (CCTCC; Wuhan City, China), using morphology and PCR assays.
Mycoplasma contamination	Cell lines were not tested for mycoplasma contamination.
Commonly misidentified lines (See ICLAC register)	No commonly misidentified lines were used.

Animals and other research organisms

Policy information about studies involving animals; ARRIVE guidelines recommended for reporting animal research, and Sex and Gender in Research

Laboratory animals	Young (3 weeks old), adult (6 - 8 weeks old) and 65 old (9 months old) C57BL/6 (Vital River Laboratories, China) mice were used in the study. Mouse housing condition: a light cycle of 12 light/12 dark was used; Temperature and humidity: Temperatures of ~18-24°C with 40-60% humidity.<br>A total of six female rhesus monkeys (3 - 5 years old, 4.65 ± 0.79 kg body weight) were pursed from SAFE Medical Technology, China and were used in this study. Monkey housing condition: a light cycle of 12 light/12 dark was used; Temperature and humidity: Temperatures of ~24-26°C with 50-60% humidity.
Wild animals	The study did not involve wild animals.
Reporting on sex	To examine the effect of sex on m6A levels, we constructed the female mice gamma-ray TBI model. A total of 65 adult (6 - 8 weeks old) female C57BL/6 mice were divided into 13 groups, including one sham-exposed group and 12 groups with different doses of gamma rays (2, 4, and 6.5 Gy) at multiple time points (day 1, 7, 14, and 28) after irradiation.
Field-collected samples	The study did not involve samples collected from the field.
Ethics oversight	Medical Ethical Committee of Beijing Institute of Radiation Medicine (Beijing, China)

Note that full information on the approval of the study protocol must also be provided in the manuscript.
