# Supplementary material for: RNA N6-methyladenosine modification-based biomarkers for absorbed ionizing radiation dose estimation
Source: Nat Commun. 2023 Oct 30;14:6912. doi: 10.1038/s41467-023-42665-w (PMC10616291; doi:10.1038/s41467-023-42665-w)
Supplement: Supplementary file 3 — Description of Additional Supplementary Files [file 41467_2023_42665_MOESM3_ESM.pdf]

Title: Supplementary Data 1.

Description: The primers used for MeRIP-qPCR and qRT-PCR assays.

Title: Supplementary Data 2.

Description: Overview of different irradiation models of mice and 58 monkeys.

Title: Supplementary Data 3.

Description: Overview of different treatments models of HUVECs and 60 human peripheral whole blood cells.

Title: Supplementary Data 4.

Description: The characteristics of healthy volunteers, radiation workers 62 and cancer patients receiving fractionated radiotherapy.

Title: Supplementary Data 5.

Description: Overview of human peripheral whole blood samples used for 64 dose-response evaluation.

Title: Supplementary Data 6.

Description: Dataset used for construction of dose prediction models in 66 TBI-treated mice.

Title: Supplementary Data 7.

Description: Dataset used for construction of dose prediction models in 68 cancer patients receiving fractionated radiotherapy
